# Unraveling the Connection between Fatty Liver Severity with Gender, Lifestyle, and Health Risks among Workers

**DOI:** 10.3390/nu15224765

**Published:** 2023-11-13

**Authors:** Feng-Cheng Tang, Ren-Hau Li, Jui-Hua Huang

**Affiliations:** 1 Department of Occupational Medicine, Changhua Christian Hospital, Changhua 500, Taiwan; 106159@cch.org.tw; 2 Department of Psychology, Chung Shan Medical University, Taichung 402, Taiwan; davidrh@csmu.edu.tw; 3 Department of Golden-Ager Industry Management, Chaoyang University of Technology, Taichung 413, Taiwan

**Keywords:** fatty liver, gender, lifestyle, metabolic anomalies, inflammation, liver dysfunction, worker

## Abstract

The purpose of this study was to investigate the correlation between the severity of fatty liver and factors such as gender, lifestyle, and the risks of metabolic abnormalities, inflammation, and liver dysfunction in the working population. This cross-sectional study included 2936 workers aged 21–64 years. The severity of fatty liver was assessed using ultrasound. A self-administered survey was used to evaluate lifestyle habits. Data on anthropometric measurements, blood pressure, blood tests, and biochemical evaluations was collected. The 45.5% of workers had fatty liver. Males had a higher prevalence of fatty liver and health risks and several unhealthy lifestyle habits compared to females. The health behavior score related to exercise showed notable declines as the severity of fatty liver increased (*p* < 0.001). Percentages of current alcohol drinkers differed among different levels of fatty liver, with rates of 43.1, 48.4, 44.8, and 63.4% (*p* = 0.005) observed in the absence, mild, moderate, and severe fatty liver, respectively. Workers with fatty liver showed increased risks related to metabolic anomalies, especially in severe cases. The risk of inflammation and liver dysfunction also significantly increased with elevated fatty liver severity. Overall, fatty liver presents significant health risks, with nearly half of the workers diagnosed with the condition. To improve liver health, it is crucial to have customized strategies for promoting health, taking into account the different levels of severity in fatty liver.

## 1. Introduction

A significant number of people worldwide are affected by fatty liver, a prevalent condition [1,2]. Worldwide, approximately one-third of adults suffer from nonalcoholic fatty liver disease (NAFLD) [3]. The prevalence of NAFLD in Asia is around 30% [2]. A nationwide Taiwan health screening database with participants aged 40–64 years showed 28% had non-alcoholic fatty liver disease (NAFLD) [4]. A retrospective study in Taiwan with subjects aged 17–65 years showed 52% had fatty liver (26% mild, 22.6% moderate, and 3.8% severe) [5]. This can lead to a higher chance of developing multisystemic illnesses, such as cardiovascular incidents, metabolic abnormalities, and kidney issues [6]. Furthermore, it has the potential to advance to cirrhosis and its associated complications, such as acute hepatitis and hepatocellular carcinoma [7]. Although it has been acknowledged as a notable health issue, there are still unresolved inquiries about the extent of its seriousness in relation to variables such as sex, personal lifestyle choices, and the resulting dangers of metabolic abnormalities, inflammation, and liver malfunction. Judging from the prevalence of fatty liver disease in Taiwan [4,5], fatty liver disease has become a liver disease that Taiwan needs to pay attention to. Specifically, the nuances of how these factors interplay among working populations in Taiwan still need to be fully understood.

Fatty liver disease arises from a combination of metabolic, genetic, epigenetic, and lifestyle influences [6,8]. The development and advancement of fatty liver are heavily influenced by lifestyle factors [9]. Individuals with genetic susceptibility, when exposed to an unhealthy diet and a sedentary lifestyle, are at risk of developing adiposity, fatty accumulation, and fatty liver disease [6,8,10]. The consumption of alcohol, even in moderate amounts, has been linked to worsened liver conditions [11,12]. Furthermore, the correlation between the occurrence of fatty liver and gender has been gaining more recognition [10,13]. Differences in lifestyle may contribute to the higher occurrence of fatty liver disease in men, as suggested by multiple studies [8,13]. Nevertheless, there is a restricted comprehension regarding the association among gender, lifestyle aspects, and the severity of fatty liver. The targeted study of these relationships in Taiwanese workers, that may have unique lifestyle [14,15], also needs to be explored.

The pathophysiological aspects of fatty liver disease, metabolic syndrome, and type 2 diabetes are closely related, with inflammatory processes in the adipose tissue, gut, and liver playing a crucial role [16]. Experts have recently suggested the term ‘metabolic dysfunction associated fatty liver disease’ (MAFLD) as a replacement for ‘NAFLD’ in order to more accurately represent the pathophysiology and cardiometabolic consequences of this prevalent liver condition [4,17]. Furthermore, contemporary research highlights the diverse associations between fatty liver and numerous health hazards [18,19]. Metabolic abnormalities can arise from fatty liver, which can contribute to the high occurrence of Type 2 diabetes and central obesity. Furthermore, there exists a reciprocal connection [19,20]. At the same time, studies have demonstrated that this condition can raise the likelihood of cardiovascular disorders [4,18], emphasizing its extensive health consequences. Although there is a general comprehension of the causes and hazards associated with fatty liver, it is necessary to adopt a more specific and focused approach to comprehend the health risks faced by workers with varying degrees of fatty liver. This approach should particularly consider metabolic risk factors, inflammatory markers, and liver functional parameters.

The current studies mainly concentrate on the general public or particular groups of patients [21,22,23]. Furthermore, the majority of studies investigate the demographic and clinical traits of individuals who have or do not have fatty liver [21,22,23]. The exploration of the correlation between the severity of fatty liver and gender, lifestyle, and related health risks among Taiwanese workers has been insufficiently studied. This represents a significant research issue in our current understanding and emphasizes the need for detailed exploration. A more nuanced comprehension could lead to effective preventive measures and targeted interventions for this specific group. Hence, the primary objective of this research is to examine and compare the hazards associated with inflammation, liver complications, and metabolic disorders among employees with varying degrees of fatty liver. Using ultrasound to evaluate the extent of fatty liver, examine participants’ lifestyle habits related to health, and analyze metabolic risk factors, markers of inflammation, and parameters of liver function. Additionally, this study will use logistic regression to offer adjusted odds ratios (ORs) and 95% confidence intervals (CI) for the mentioned health risks related to the severity of fatty liver. This will provide valuable insights in the field.

## 2. Materials and Methods

### 2.1. Ethical Committee Review

Under the regulations of the Taiwanese Labor Health Protection Rule, it is mandatory for occupational health professionals to periodically evaluate the health hazards and requirements of employees. As a component of the Taiwan Workplace-Health-Promotion Scheme, this research was carried out through the implementation of public health monitoring among employees. Each candidate was given an explanation of the study through an accompanying information sheet that came with the questionnaire. Participants had the freedom to choose whether or not they wanted to take part in this research. Volunteer participants were asked to fill out a questionnaire. Returned questionnaires, along with participants’ clinical variables, were coded by occupational health personnel at each contact company. Hence, in terms of investigation, all information gathered in the research is maintained anonymous and strictly confidential to safeguard the participants’ privacy. The research was carried out following the guidelines of the Declaration of Helsinki and received approval from the Institutional Review Board of Changhua Christian Hospital in Taiwan, with a waiver of informed consent (approval number CCH IRB 191238).

### 2.2. Design and Participants of the Study

Cross-sectional research with convenience sampling was used for this study. Three industrial enterprises in central Taiwan recruited employees 20 years of age or older to take part in the study. Manufacturing of electronic components, auto parts, and transportation equipment were among their main pursuits. The businesses were picked because of their positive interactions with the Center for Occupational Health. This made it possible for the investigation to move along smoothly. The 4107 workers in all were voluntarily participants in the study.

In Taiwan, fewer women work than those in the United States and the like, so the male ratio was higher in our study. However, this study performed the chi-square goodness of fit test for the distribution ratio between the male (*n* = 3435, 83.6%) and female (*n* = 672, 16.4%) of the 4107 workers and the sample with the male (*n* = 2469, 84.1%) and female (*n* = 467, 15.9%) of the 2936 workers. According to the test, the *p* value was 0.470, which did not reach statistical significance. It is obvious that the sample data fits the distribution of the population of the three companies.

### 2.3. Assessment of Health-Related Lifestyle Habits

A self-administered survey was used to evaluate the health-related lifestyle habits of the participants, which encompassed aspects such as dietary choices, physical activity, smoking, and alcohol intake.

We acquired information on dietary and physical activity habits by utilizing subsets of the Health Promoting Lifestyle Profile II [24,25]. The nutrition behavior category includes nine elements, while the exercise behavior category includes eight elements. Both categories are assessed using a four-point Likert scale, which includes the options of Never, Sometimes, Often, and Routinely. The average score for each subscale, which ranged from 1 to 4, was computed by dividing the total score of the subscale by the number of response items. A higher rating indicated a higher degree of engagement in behavior that promotes health. The nine inquiries concerning dietary habits were “select a low-fat diet”, “restrict the consumption of sugars”, “consume bread, cereal and rice”, “consume fruit”, “consume vegetables”, “consume meat, poultry, fish, legumes, eggs and nuts”, “consume milk, yogurt or cheese”, “examine labels to determine nutrients” and “have breakfast”. Dietary guidelines determined the recommended daily servings for each food category. The exercise behavior checklist includes the following eight items: “adhere to an exercise program”, “engage in intense physical activity three times per week”, “participate in light to moderate physical activities”, “attend exercise sessions during leisure time”, “perform stretching exercises three times per week”, “incorporate physical activity into daily routines”, “monitor pulse while exercising”, and “achieve a desired heart rate during exercise”. These items were translated into traditional Chinese characters for the participants. The internal consistency of the nutrition and exercise behavior subscales in the Taiwanese population was found to be satisfactory, with Cronbach’s alpha values of 0.78 and 0.85, respectively, according to the findings of this research.

Furthermore, the smoking status of every participant was categorized as non-smokers and current smokers (which includes both occasional and daily smoking). The consumption of alcohol was categorized into two groups: non-alcohol drinkers and current alcohol drinkers (which includes occasional or daily drinking).

### 2.4. Fatty Liver Severity Determination

The severity of fatty liver was assessed using ultrasound images [26]. After undergoing ultrasound examinations, workers were graded as follows: (0) absent: echotexture of the right liver lobe is normal compared to cortex of the right kidney; (1) mild: slightly and diffusely increased liver echogenicity with normal visualization of portal vein wall and diaphragm; (2) moderate increase of liver echogenicity with slightly impaired visualization of the portal vein wall and the diaphragm and (3) severe: marked increase of liver echogenicity with poor or non-visualization of portal vein wall, diaphragm, and posterior right lobe of the liver [27,28]

### 2.5. Evaluation of Metabolic Risk Factors

Data on metabolic risk factors including waist circumference, systolic blood pressure (SBP) and/or diastolic blood pressure (DBP), fasting blood glucose (FBG), triglyceride levels, and high-density lipoprotein cholesterol (HDL-C) levels were gathered. As per the Health Promotion Administration’s definition, provided by the Ministry of Health and Welfare in Taiwan [29], the metabolic syndrome is determined by the following criteria: (1) Abdominal obesity, where waist circumference is equal to or greater than 90 cm (35 inches) for men and equal to or greater than 80 cm (31 inches) for women. (2) Elevated blood pressure with SBP greater than or equal to 130 mmHg or DBP greater than or equal to 85 mmHg. (3) Elevated FBG levels with a value of 100 mg/dL or higher. Elevated levels of triglycerides during fasting, equal to or greater than 150 mg/dL. Men with HDL-C levels below 40 mg/dL and women with levels below 50 mg/dL are considered to have low HDL-C. Metabolic syndrome can be definitively identified if the aforementioned five components satisfy three or more criteria [29].

### 2.6. Inflammatory and Cardiovascular Markers Detection

White blood cell (WBC) and platelet count assays were employed to identify markers associated with inflammation and cardiovascular health [30,31,32]. Because the subjects of this study are general workers and not cases of acute inflammation. Therefore, the following method is used to divide the thresholds of inflammation indicators: the participants were divided into three groups (low, medium, and high) using stratification into tertiles. High levels of the two inflammatory markers, WBC count exceeding 7.16 (10^9^/L) and platelet count surpassing 270 (10^9^/L), are defined as such.

### 2.7. Liver Functional Parameters Measurement

Liver functional parameters were assessed by measuring glutamate oxaloacetate transaminase (GOT) and glutamate pyruvate transaminase (GPT). The hospital medical laboratory reported that the GPT reference range was between 0 and 35 U/L. The reference range of GOP was 5~40 U/L.

### 2.8. Statistical Analysis

The Shapiro-Wilk test indicated that most of the continuous dependent variables were significant, which meant non-normal distribution”. However, according to Hair et al. (2010) and Bryne (2010), data is can be viewed as normal if the skewness is between −2 and +2 and the kurtosis is between −7 and +7 [33,34]. The deviation of data from normality was not severe as skewness and kurtosis index were below 3 and 10 respectively [35]. The examination of association between two categorical variables is conducted using the Chi-square test. Data are number (*n*), percent (%). A t-test was used to compare the averages of two different groups. We compared the averages of four groups by conducting a one-way ANOVA and then performing Scheffe’s multiple comparisons test. After comparing the means of four groups, ANOVA trend analyses were conducted using line by polynomial contrasts. Data are means (95% Confidence Interval for Mean). The relationship between levels of fatty liver (as an independent variable) and risk factors related to inflammation, cardiovascular health, liver function, and metabolism (as a dependent variable respectively) were analyzed using logistic regression models. The adjusted odds ratio (95% confidence intervals) is calculated for the data. All statistical procedures were performed using statistical software IBM SPSS version 22 (SPSS Inc., Chicago, IL, USA), and a statistically significant at *p* < 0.05.

## 3. Results

### 3.1. Characteristics of Participants by Gender

Table 1 displayed the gender-based characteristics of the participants. In this study, 4107 workers (83.6% male), aged 20 or over, were volunteered as subjects from the three companies. However, part of the workers did not provide the necessary information on personal data, health-related lifestyle habits, or a physical examination, and therefore were excluded. A final number of 2936 workers (84.1% male) with an average age of 42.5 years were enrolled. Out of the total, 45.5% were found to have fatty liver, with the levels of severity classified as mild (34.3%), moderate (9.8%), and severe (1.4%). Furthermore, males exhibited a greater prevalence of fatty liver (48.5%) in comparison to females (29.3%, *p* < 0.001). In addition, male participants had higher rates of current smokers and current alcohol drinkers and showed worse nutritional habits compared to their female counterparts (*p* < 0.001). Nevertheless, there were no notable disparities in physical activity habits between males and females.

### 3.2. Correlation between the Lifestyle Habits and Fatty Liver Levels

Individuals with fatty liver had a higher prevalence of smoking (*p* < 0.011) and displayed inferior dietary habits (*p* < 0.019) and physical activity habits (*p* < 0.001) compared to those without fatty liver.

In addition, according to Table 2, to examine the differences between the averages of four different levels of fatty liver, a one-way analysis of variance (ANOVA) was conducted. The findings showed statistically significant differences in the exercise health behavior score (*p* < 0.001). Nevertheless, there were no notable disparities detected in dietary habits among different stages of fatty liver. Furthermore, this research discovered that the proportion of current alcohol drinkers differed among employees, with percentages of 45.0, 48.4, 44.8, and 63.4% (*p* = 0.048) observed in the absence, slight, moderate, and intense fatty liver conditions, respectively. There were slight variations in smoking among different levels of fatty liver (*p* = 0.062).

### 3.3. Mean or Percentage of Metabolic Parameters, Inflammatory Markers, and Liver Functional Parameters by Fatty Liver Levels

**Figure 1:**
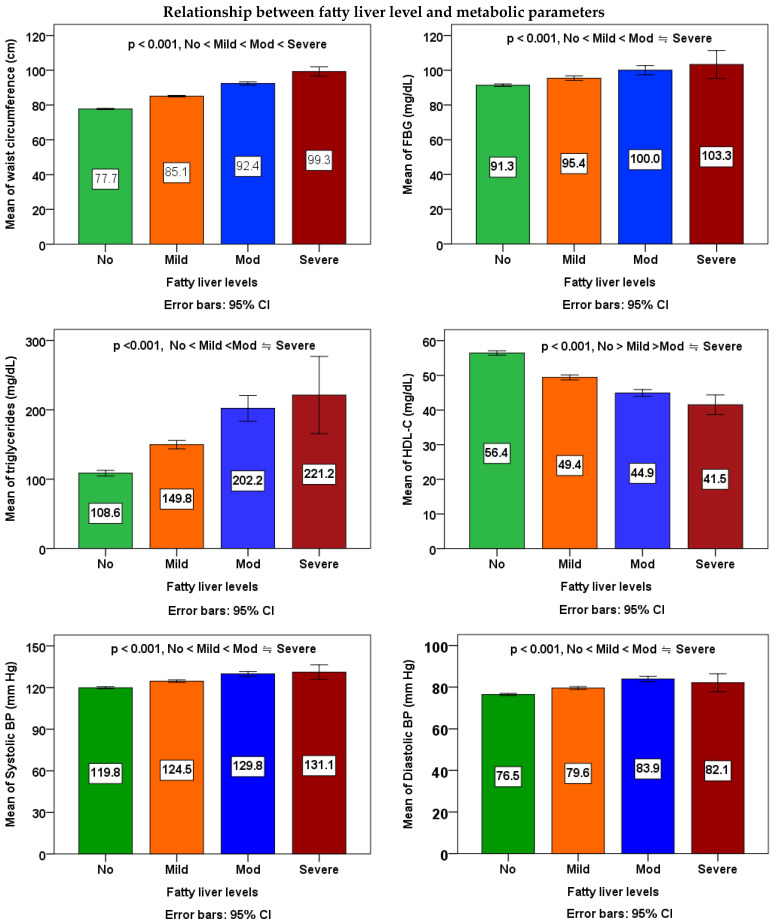

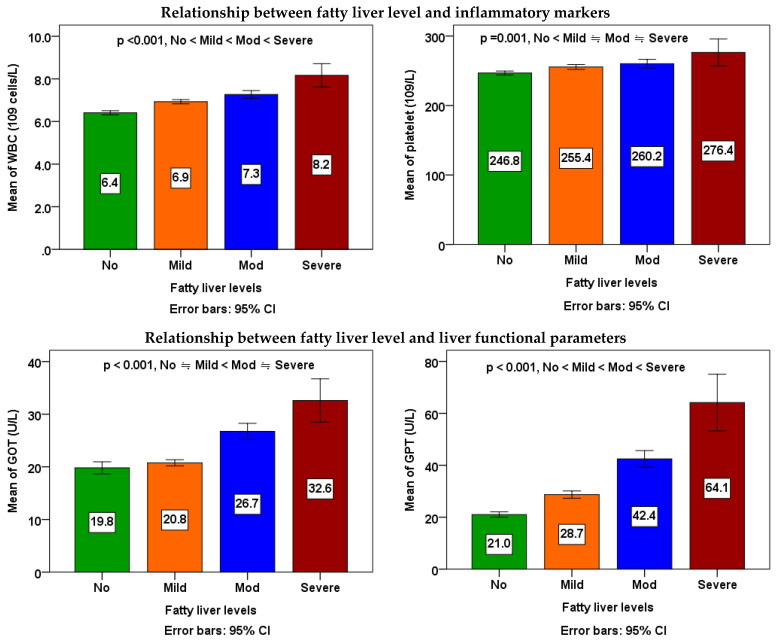
Relationship between fatty liver levels and metabolic parameters, inflammatory markers, and liver functional parameters. The means of four groups were compared using one-way ANOVA followed by Scheffe’s multiple comparisons test. A *p* value of <0.05 was considered statistically significant.

According to Table 3 and Figure 1, to examine the differences between the averages of four different levels of fatty liver, a one-way analysis of variance (ANOVA) was conducted. The mean of each metabolic parameters, inflammatory markers, and liver functional parameters in the four different fatty liver subgroups among workers was significantly different (*p* < 0.001). Workers with fatty liver may have a significant propensity toward the mean of each metabolic parameters, inflammatory markers, and liver functional parameters when compared to those without the condition. In Scheffe’s post-hoc comparison, waist circumference, WBC, and GPT showed a salient trend (*p* < 0.001); the data were increased along with higher severity of fatty liver. For FBG, triglycerides, HDL-C, systolic BP, and diastolic BP, all of the differences between any two subgroups were also significant, except those between the mod subgroup and the severe subgroup. For platelets, differences between non-fatty liver subgroups and the mild subgroup, mod subgroup, and severe subgroup were significant, except those between the mild subgroup, mod subgroup, and severe subgroup. For GOT, all of the differences between any two subgroups were also significant, except those between the non-fatty liver subgroup and the mild subgroup, as well as between the mod subgroup and the severe subgroup.

The ANOVA linear trend analyses demonstrated noteworthy rises in waist size, FBG, triglycerides, systolic BP, and diastolic BP associated with higher severity of fatty liver (*p* for linear trend < 0.001), while indicating a decline in HDL cholesterol levels (*p* for linear trend < 0.001). A notable rise in WBC count (*p* for linear trend < 0.001) and platelet count (*p* for linear trend = 0.001) in correlation with increased severity of fatty liver. Furthermore, a notable rise in the count of GOT and GPT with increased severity of fatty liver (*p* for linear trend < 0.001).

### 3.4. The Odds Ratio of Metabolic Irregularity, Inflammation, and Liver Impairment Based on the Severity of Fatty Liver

Fatty liver severity determines the adjusted odds ratio (OR) of metabolic abnormality, inflammation, and liver dysfunction, as depicted in Table 4. Compared to workers who do not have fatty liver, those with mild, moderate, and severe fatty liver were significantly linked to a higher risk of obesity (*p* < 0.001), particularly severe fatty liver (OR 270.0). Employees who have mild, moderate, or severe fatty liver also exhibited a considerably greater likelihood of experiencing high FBG, elevated triglyceride levels, increased systolic BP, elevated diastolic BP, and reduced HDL-C levels in comparison to individuals without fatty liver (*p* < 0.001).

Workers with different levels of fatty liver severity showed an increased risk of elevated WBC count (OR 1.6, 2.7, and 5.5) and elevated platelet count (OR 1.4, 1.9, and 2.5) compared to workers without fatty liver.

Workers who had mild, moderate, or severe fatty liver also showed an increased likelihood of having high GPT levels (with odds ratios of 2.3, 8.4, and 28.3, respectively) compared to those without fatty liver. Furthermore, individuals with moderate and severe hepatic steatosis exhibited a notably elevated likelihood of elevated GOT levels (with odds ratios of 3.7 and 16.9, respectively) compared to those without fatty liver.

In the odds ratio comparison between the four different fatty liver subgroups, obese, high FBG, metabolic syndrome, and high GPT showed a salient trend (*p* < 0.001); the data increased along with the severity of fatty liver. For high triglycerides, low HDL, high BP, high WBC, and high platelets, all of the differences between any two subgroups were also significant, except those between the mod subgroup and the severe subgroup. For high GOT, all of the differences between any two subgroups were also significant, except those between the non-fatty liver subgroup and the mild subgroup.

## 4. Discussion

The main findings of this research are as follows: (1) A large number of Taiwanese workers, specifically 45.5% of them, were found to have fatty liver; (2) The prevalence of fatty liver and health risks was higher among male workers who also had several unhealthy lifestyle habits compared to females; (3) There were noticeable connections between the severity of fatty liver and lifestyle habits like alcohol consumption and lack of exercise; and (4) As the severity of fatty liver increased, there was a clear pattern of metabolic abnormalities, inflammation, and liver dysfunction, particularly in severe cases.

### 4.1. The Prevalence of Fatty Liver Disease

The Non-Alcoholic Fatty Liver Disease (NAFLD) is a significantly widespread issue in public health, affecting approximately 32% of the adult population worldwide [2]. Approximately 30% [3,36] of individuals in Asia are affected by NAFLD, indicating its high prevalence in the region. A nationwide Taiwan health screening database with participants aged 40–64 years showed 28% had non-alcoholic fatty liver disease (NAFLD) [4]. A retrospective study in Taiwan with subjects aged 17–65 years showed 52% had fatty liver (26% mild, 22.6% moderate, and 3.8% severe) [5]. Furthermore, research indicated that the prevalence of NAFLD was 48.4% among the working population in the northern region of Taiwan [37]. Among workers in central Taiwan, the present study revealed that the prevalence of fatty liver was 45.5%. Based on the data provided, the significant occurrence of fatty liver among employees in Taiwan is a matter that deserves attention.

Due to its association with a higher susceptibility to various health issues such as cardiovascular events, metabolic disorders, and renal problems [6], fatty liver can lead to an elevated risk of multisystem disease. Furthermore, it has the potential to advance to cirrhosis and its associated complications, such as acute hepatitis and hepatocellular carcinoma. Hence, it is essential to implement efficient measures targeting the reduction of fatty liver occurrence among employees, as it has the potential to enhance their overall well-being. If health promoters do not address this growing health problem, unhealthy workers may in turn have an impact on business as well, reducing the nation’s productivity.

### 4.2. Gender Differences in Fatty Liver Status and Lifestyle Habits

Gender, age, and metabolic dysfunction differences are among the additional elements that impact the progression of NAFLD [13]. NAFLD shows marked differences in prevalence and severity with regards to gender. According to estimated global prevalence of NAFLD, it is greater in males (40%) than in females (26%) [2]. Males exhibited a greater prevalence of fatty liver (48.5%) in contrast to females (29.3%) according to the findings of the present research. According to a study, Chinese occupational population in Taipei, Taiwan showed a higher occurrence of NAFLD in males compared to females (57.8% vs. 32.4%) [37]. In a cross-sectional investigation in the northeast of Thailand, the occurrence of NAFLD among females was 22.9%, whereas males had a lower rate of 18.3% [38].

The prevalence of NAFLD may be influenced by factors such as the impact of variations in lifestyles and physiology [13,39], leading to gender differences observed in NAFLD [13]. According to this research, the occurrence of fatty liver disease is more common among males, possibly due to variations in their way of living. Male workers reported higher rates of smoking and alcohol consumption and exhibited poorer nutritional habits than female workers. Indeed, health behaviors were different between men and female. Several studies indicated that women exhibit greater engagement in general health-enhancing lifestyles or specific health-promoting actions [40,41]. The above-mentioned results indicate that when designing workplace health promotion programs, it is important to take into account the varying health behaviors of men and women in order to minimize the likelihood of developing fatty liver. Nevertheless, numerous elements play a role in the variation of fatty liver occurrence between genders, necessitating additional research in the future to gain a deeper comprehension of the underlying mechanisms and potential treatment options.

### 4.3. The Correlation between the Severity of Fatty Liver and Lifestyle Behaviors

The development and advancement of fatty liver are heavily influenced by lifestyle factors [42]. Nevertheless, the knowledge regarding the association between fatty liver severity and lifestyle factors remains restricted. The present study unveiled that individual with hepatic steatosis displayed elevated smoking rates and demonstrated inferior dietary and physical activity habit. It is important to mention that this study uncovered notable reductions in the score of exercise health behavior as the severity of fatty liver increased. Moreover, there is a notable distinction between the groups with no fatty liver and moderate fatty liver, as well as between the groups with no fatty liver and severe fatty liver. The distinction between the groups with no fatty liver and mild fatty liver exhibited only marginal significance. This may mean that workers with moderate to severe fatty liver disease may have a significant lack of exercise. In addition, the relationship between healthy nutritional behaviors and fatty liver severity was not significant in this study. Nonetheless, patients with fatty liver disease had apparent worse eating habits than those without fatty liver disease. On the other hand, the proportion of alcohol intake differed among various levels of fatty liver, with a particularly notable increase in cases of severe fatty liver (45.0% for no fatty liver, 48.4% for mild fatty liver, 44.8% for moderate fatty liver, and 63.4% for severe fatty liver).

A case-control study revealed that a stronger commitment to a healthy lifestyle, as indicated by a higher score in healthy lifestyle factors, was linked to a reduced risk of NAFLD [43]. According to the 2016–2017 Chilean National Health Survey, a reduced prevalence of smoking, increased engagement in moderate-vigorous physical activity, and higher consumption of whole-grains were linked to a decreased likelihood of developing NAFLD [23]. Furthermore, the consumption of alcohol, even in moderate amounts, has been linked to worsened liver conditions [11,12]. It is evident from the above that implementing health promotion strategies that target the promotion of particular lifestyle behaviors could be beneficial in enhancing the severity of fatty liver.

### 4.4. The Correlation between the Severity of Fatty Liver and Metabolic Abnormalities, Inflammation, and Liver Dysfunction

According to multiple reports, NAFLD is a disease that affects multiple systems and greatly raises the likelihood of inflammation outside the liver and chronic cardiometabolic disorders [1,6,19]. The above-mentioned diverse non-liver ailments encompassing heart disease, diabetes type 2, long-term kidney disease, and specific non-liver cancers [1,6,19].

Lu et al., conducted a comprehensive review and meta-analysis investigated the correlation between obesity and NAFLD, emphasizing the link between liver performance and metabolic disorder [44]. Moreover, the correlation between fatty liver and markers of inflammation contributes to the increasing body of evidence connecting liver impairment with systemic inflammation [16,45]. Adams et al. explored the essential physiological roles of the liver in glucose and lipid metabolism and examined how these functions can be disrupted in NAFLD, potentially resulting in systemic inflammation [45]. However, the knowledge regarding the connection between the severity of fatty liver and the risk of metabolic abnormalities, inflammation, and liver dysfunction remains restricted.

In the present study, it was discovered that as the severity of fatty liver disease increases, the risks associated with metabolic abnormalities, inflammation, and liver dysfunction increase, especially in severe cases. These risks include higher waist circumference, triglycerides, blood pressure, and lower HDL cholesterol levels. Markers of inflammation (white blood cell and platelet counts) and liver function parameters (GOT and GTP) also increased significantly with the increasing severity of fatty liver disease. Likewise, a prior investigation suggested that the prevalence of diabetes mellitus, metabolic syndrome, and susceptibility to cardiovascular disease rose in tandem with the escalating severity of fatty liver, as reported in a different study [46]. Metabolic abnormalities and inflammation are factors that impact fatty liver disease and, in this sense, may predict the severity of the disease, increasing its risk. Individuals with severe liver disease, compared to those with a mild or moderate condition, are characterized by a worse cardiometabolic profile, which in turn increases the risk of progression.

### 4.5. Achievements and Implications

The primary significance of this research lies in its thorough investigation of the severity of fatty liver in employees, with a particular emphasis on metabolic risk factors, markers of inflammation, and parameters related to liver function. The present study offers valuable information on the occurrence and dangers linked to different levels of fatty liver by utilizing a cross-sectional approach and statistical techniques. The results emphasize the significance of identifying the problem at an early stage and implementing customized measures to reduce the potential dangers linked to this widespread ailment.

### 4.6. Limitations and Prospective

Although the present study has made valuable contributions, it is not without its limitations. Firstly, the observed risks are limited in their ability to establish causality between fatty liver severity and the cross-sectional design. Secondly, the potential for bias may arise due to the dependence on self-reported questionnaires to assess lifestyle habits. Third, this study used convenience sampling, which is a non-probability sampling technique. Therefore, the study results are not necessarily generalizable. Future research should consider longitudinal designs, incorporate objective measures of lifestyle habits, and expand the sample to diverse populations. Additionally, future exploration will be crucial in investigating the underlying mechanisms that connect the severity of fatty liver with the observed risks and exploring targeted interventions.

## 5. Conclusions

The current analysis explains the important discoveries of the present study, situating them within the framework of previous studies and emphasizing their scholarly contributions. The identified limitations and future research directions pave the way for continued investigation into this critical area of liver health. The results of this study highlight the immediate requirement for customized health-promotion tactics, taking into account the different levels of fatty liver severity, in order to improve the liver well-being of employees and the broader population.

## Figures and Tables

**Table 1:** Characteristics of participants by gender ^1^.

		Gender		
Variables	Total (*n* = 2936)	Male (*n* = 2469)	Female (*n* = 467)	*p*
Age (y)	42.5 ± 10.0	41.8 ± 10.2	46.0 ± 7.4	<0.001
Lifestyle habits				
Nutritional health behavior	2.5 ± 0.4	2.4 ± 0.4	2.7 ± 0.4	<0.001
Exercise health behavior	2.0 ± 0.6	1.9 ± 0.6	2.0 ± 0.6	0.344
Smoking				
Current smokers	656 (22.3)	654 (26.5)	2 (0.4)	<0.001
Non-smokers	2280 (77.7)	1815 (73.5)	465 (99.6)	
Alcohol consumption				
Current alcohol drinkers	1363 (46.4)	1265 (51.2)	98 (21.0)	<0.001
Non-alcohol drinkers	1573 (53.6)	1204 (48.8)	369 (79.0)	
Fatty liver levels				
No	1601 (54.5)	1271 (51.5)	330 (70.7)	<0.001
Mild	1006 (34.3)	892 (36.1)	114 (24.4)	
Mod	288 (9.8)	267 (10.8)	21 (4.5)	
Severe	41 (1.4)	39 (1.6)	2 (0.4)	

^1^ *p*-value less than 0.05 was considered statistically significant. Continuous data are presented in mean ± SD. Categorical data are presented in number (*n*) and percent (%).

**Table 2:** Correlation between the lifestyle habits and fatty liver levels ^1,2^.

	Fatty Liver Levels				
Variables	No (*n* = 1601)	Mild (*n* = 1006)	Mod (*n* = 288)	Severe (*n* = 41)	*p*
Nutritional health behavior	2.48 (2.45–2.50)	2.43 (2.41–2.46)	2.46 (2.41–2.50)	2.44 (2.31–2.57)	0.104
Exercise health behavior	1.99 (1.96–2.02)	1.93 (1.90–1.96)	1.86 (1.81–1.92)	1.74 (1.55–1.92)	<0.001
Smoking					
Current smokers	329 (20.5)	252 (25.0)	65 (22.6)	10 (24.4)	0.062
Non-smokers	1272 (79.5)	754 (75.0)	223 (77.4)	31 (75.6)	
Alcohol consumption					
Current alcohol drinkers	721 (45.0)	487 (48.4)	129 (44.8)	26 (63.4)	0.048
Non-alcohol drinkers	880 (55.0)	519 (51.6)	159 (55.2)	15 (36.6)	

^1^ The means of four groups were compared using one-way ANOVA. Data are means (95% Confidence Interval for Mean). Statistically significant at *p* < 0.05. ^2^ The Chi-square test is used to examine association between two categorical variables. Data are number (*n*), percent (%). Statistically significant at *p* < 0.05.

**Table 3:** Mean or percentage of metabolic parameters, inflammatory markers, and liver functional parameters by fatty liver levels ^1,2^.

	Fatty Liver Levels				ANOVA ^1^ F, *p*	*p* for Linear Trend ^1^
Variables ^3^	No (*n* = 1601)	Mild (*n* = 1006)	Mod (*n* = 288)	Severe (*n* = 41)		
Metabolic parameters						
Waist circumference (cm) ^a,b,c,d,e,f^	77.7 (77.3–78.1)	85.1 (84.6–85.5)	92.4 (91.4–93.3)	99.3 (96.6–101.9)	471.3, <0.001	<0.001
FBG (mg/dL) ^a,b,c,d^	91.3 (90.6–92.1)	95.4 (94.1–96.8)	100.0 (97.4–102.7)	103.3 (95.3–111.3)	26.2, <0.001	<0.001
Triglycerides (mg/dL) ^a,b,c,d,e^	108.6 (104.6–112.6)	149.8 (143.7–155.9)	202.2 (183.7–220.7)	221.2 (165.6–276.9)	99.8, <0.001	<0.001
HDL-C (mg/dL) ^a,b,c,d^	56.4 (55.8–57.0)	49.4 (48.7–50.1)	44.9 (43.9–45.9)	41.5 (38.6–44.4)	128.8, <0.001	<0.001
Systolic BP (mm Hg) ^a,b,c,d^	119.8 (119.1–120.5)	124.5 (113.6–125.5)	129.8 (128.0–131.5)	131.1 (125.9–136.3)	50.7, <0.001	<0.001
Diastolic BP (mm Hg) ^a,b,c,d^	76.4 (75.9–77.0)	79.6 (78.9–80.2)	83.9 (82.5–85.2)	82.1 (77.8–86.5)	45.9, <0.001	<0.001
Metabolic syndrome						
Yes (*p* < 0.001)	70 (4.4)	175 (17.4)	123 (42.7)	26 (63.4)		
No	1531 (95.6)	831 (82.6)	165 (57.3)	15 (36.6)		
Inflammatory markers ^1^						
WBC (10^9^ cells/L) ^a,b,c,d,e,f^	6.4 (6.3–6.5)	6.9 (6.8–7.0)	7.3 (7.1–7.5)	8.2 (7.6–8.7)	38.6, <0.001	<0.001
Platelet (10^9^/L) ^a,b,c^	246.8 (243.9–249.6)	255.4 (251.9–258.9)	260.2 (254.0–266.4)	276.4 (257.0–295.8)	10.1, <0.001	0.001
Liver functional parameters						
GOT (U/L) ^b,c,d,e^	20.2 (18.7–20.9)	20.8 (20.2–21.3)	26.7 (25.2–28.3)	32.6 (28.5–36.7)	16.7, <0.001	<0.001
GPT (U/L) ^a,b,c,d,e,f^	21.7 (20.0–22.0)	28.7 (27.3–30.1)	42.4 (39.2–45.6)	64.1 (53.2–75.1)	120.6, <0.001	<0.001

^1^ The means of four groups were compared using one-way ANOVA followed by Scheffe’s multiple comparisons test. Additionally, conducting ANOVA trend analyses using line by polynomial contrasts after a difference between the means of four groups were compared. Data are means (95% Confidence Interval for Mean). Statistically significant at *p* < 0.05. ^a^ indicates significant difference between no fatty liver and mild fatty liver groups. ^b^ indicates significant difference between no fatty liver and mod fatty liver groups. ^c^ indicates significant difference between no fatty liver and severe fatty liver groups. ^d^ indicates significant difference between mild fatty liver and mod fatty liver groups. ^e^ indicates significant difference between mild fatty liver and severe fatty liver groups. ^f^ indicates significant difference between mod fatty liver and severe fatty liver groups. ^2^ The Chi-square test is used to examine association between two categorical variables. Data are number (*n*), percent (%). Significant difference (*p* < 0.05). ^3^ Abbreviations: FBG, fasting blood glucose; HDL-C, high-density lipoprotein cholesterol; BP, blood pressure; WBC, white blood cell; GOT, Glutamate oxaloacetate transaminase; GPT, Glutamate pyruvate transaminase.

**Table 4:** Odds ratio of metabolic abnormality, inflammation, and liver dysfunction by fatty liver levels ^1^.

Variables ^2^	Fatty Liver Levels				Odds Comparison between Four Groups
	No (*n* = 1601)	Mild (*n* = 1006)	Mod (*n* = 288)	Severe (*n* = 41)	
Metabolic abnormality					
Waist circumference (cm) ≥90 for men or ≥80 for women (obese)	1.00	5.3 (4.2–6.7)	24.7 (18.1–33.9)	270.0 (64.1–1137.9)	No < Mild < Mod < Severe
*p*		<0.001	<0.001	<0.001	
FBG > 100 mg/dL	1.00	2.0 (1.4–2.8)	4.3 (2.8–6.5)	9.7 (4.6–20.7)	No < Mild < Mod < Severe
*p*		<0.001	<0.001	<0.001	
Triglycerides ≥ 150 mg/dL	1.00	2.7 (2.2–3.2)	5.7 (4.3–7.5)	7.0 (3.7–13.4)	No < Mild < Mod ≒ Severe
*p*		<0.001	<0.001	<0.001	
Low HDL-C	1.00	2.9 (2.3–3.6)	5.0 (3.6–6.8)	8.3 (4.3–16.0)	No < Mild < Mod ≒ Severe
*p*		<0.001	<0.001	<0.001	
BP (mm Hg) Systolic BP ≥ 130 and diastolic BP ≥ 85	1.00	1.7 (1.5–2.1)	3.8 (2.9–5.0)	4.5 (2.3–8.8)	No < Mild < Mod ≒ Severe
*p*		<0.001	<0.001	<0.001	
Metabolic syndrome	1.00	4.4 (3.3–5.9)	16.3 (11.54–22.9)	44.5 (22.1–89.6)	No < Mild < Mod < Severe
*p*		<0.001	<0.001	<0.001	
Inflammation ^3^					
WBC ≥ 7.16 × 10^9^/L	1.00	1.6 (1.4–1.9)	2.7 (2.1–3.6)	5.5 (2.8–10.9)	No < Mild < Mod ≒ Severe
*p*		<0.001	<0.001	<0.001	
Platelet ≥ 271 × 10^9^/L	1.00	1.4 (1.2–1.7)	1.9 (1.4–2.4)	2.5 (1.3–4.8)	No < Mild < Mod ≒ Severe
*p*		<0.001	<0.001	0.007	
Liver dysfunction					
GPT > 35 U/L	1.00	2.3 (1.8–2.8)	8.4 (6.2–11.3)	28.3 (12.8–62.7)	No < Mild < Mod < Severe
*p*		<0.001	<0.001	<0.001	
GOT > 40 U/L	1.00	1.2 (0.7–1.9)	3.7 (2.2–6.3)	16.9 (8.0–35.8)	No ≒ Mild < Mod < Severe
*p*		0.498	<0.001	<0.001	

^1^ Logistic regression models were used to examine the relationship between fatty liver levels (as a predictor variable) with cardiovascular, liver functional and metabolic risk factors (as a dependent variable, respectively). Adjusted confounding factors were sex, age, nutrition health behavior, exercise health behavior, smoking, and alcohol consumption. Data are odds ratio (95% CIs). Statistically significant at *p* < 0.05. ^2^ Abbreviations: FBG, Fasting blood glucose; HDL-C, high-density lipoprotein cholesterol; BP, blood pressure; WBC, white blood cell; GOT, Glutamate oxaloacetate transaminase; GPT, Glutamate pyruvate transaminase.^3^ Based on the count of WBC and platelets, the participants were divided into three groups (low, medium, and high) using stratification into tertiles. High levels of the two inflammatory markers, WBC count exceeding 7.16 (10^9^/L) and platelet count surpassing 270 (10^9^/L), are defined as such.

## Data Availability

The data are not publicly available due to privacy and ethical restrictions.

## References

[1] Fang X., Song J., Zhou K., Zi X., Sun B., Bao H., Li L. (2023). Molecular Mechanism Pathways of Natural Compounds for the Treatment of Non-Alcoholic Fatty Liver Disease. Molecules.

[2] Teng M.L., Ng C.H., Huang D.Q., Chan K.E., Tan D.J., Lim W.H., Yang J.D., Tan E., Muthiah M.D. (2023). Global incidence and prevalence of nonalcoholic fatty liver disease. Clin. Mol. Hepatol..

[3] Riazi K., Azhari H., Charette J.H., Underwood F.E., King J.A., Afshar E.E., Swain M.G., Congly S.E., Kaplan G.G., Shaheen A.A. (2022). The prevalence and incidence of NAFLD worldwide: A systematic review and meta-analysis. Lancet Gastroenterol. Hepatol..

[4] Lee H., Lee Y.H., Kim S.U., Kim H.C. (2021). Metabolic Dysfunction-Associated Fatty Liver Disease and Incident Cardiovascular Disease Risk: A Nationwide Cohort Study. Clin. Gastroenterol. Hepatol..

[5] Lin Y.C., Chou S.C., Huang P.T., Chiou H.Y. (2011). Risk factors and predictors of non-alcoholic fatty liver disease in Taiwan. Ann. Hepatol..

[6] Bilson J., Sethi J.K., Byrne C.D. (2022). Non-alcoholic fatty liver disease: A multi-system disease influenced by ageing and sex, and affected by adipose tissue and intestinal function. Proc. Nutr. Soc..

[7] Hsu C.S., Kao J.H. (2012). Non-alcoholic fatty liver disease: An emerging liver disease in Taiwan. J. Formos. Med. Assoc..

[8] Juanola O., Martinez-Lopez S., Frances R., Gomez-Hurtado I. (2021). Non-Alcoholic Fatty Liver Disease: Metabolic, Genetic, Epigenetic and Environmental Risk Factors. Int. J. Env. Res. Public Health.

[9] Farhud D.D. (2015). Impact of Lifestyle on Health. Iran J. Public Health.

[10] Rutten-Jacobs L.C., Larsson S.C., Malik R., Rannikmae K., Consortium M., International Stroke Genetics C., Sudlow C.L., Dichgans M., Markus H.S., Traylor M. (2018). Genetic risk, incident stroke, and the benefits of adhering to a healthy lifestyle: Cohort study of 306 473 UK Biobank participants. BMJ.

[11] Park J.W., Suk K.T. (2023). The effect of moderate alcohol consumption on nonalcoholic fatty liver disease. Clin. Mol. Hepatol..

[12] Weng G., Dunn W. (2019). Effect of alcohol consumption on nonalcoholic fatty liver disease. Transl. Gastroenterol. Hepatol..

[13] Nagral A., Bangar M., Menezes S., Bhatia S., Butt N., Ghosh J., Manchanayake J.H., Mahtab M.A., Singh S.P. (2022). Gender Differences in Nonalcoholic Fatty Liver Disease. Euroasian J. Hepatogastroenterol..

[14] Health Promotion Administration, Ministry of Health and Welfare in Taiwan The 4 + 1 Programs of Smart Workplaces. Innovative Services to Increase Health. https://www.hpa.gov.tw/EngPages/Detail.aspx?nodeid=1038&pid=10534.

[15] Liao Y.L.C., Park J.H. (2019). Is motorcycle use associated with unhealthy lifestyles? Findings from Taiwan. J. Transp. Health.

[16] Grander C., Grabherr F., Moschen A.R., Tilg H. (2016). Non-Alcoholic Fatty Liver Disease: Cause or Effect of Metabolic Syndrome. Visc. Med..

[17] Uehara T., Wakui H., Tamura K. (2023). Metabolic dysfunction-associated fatty liver disease reflects a significantly higher risk of hypertension than non-alcoholic fatty liver disease. Hypertens. Res..

[18] Guo Y., Yang J., Ma R., Zhang X., Guo H., He J., Wang X., Cao B., Maimaitijiang R., Li Y. (2022). Metabolic Dysfunction-Associated Fatty Liver Disease Is Associated with the Risk of Incident Cardiovascular Disease: A Prospective Cohort Study in Xinjiang. Nutrients.

[19] Godoy-Matos A.F., Silva Junior W.S., Valerio C.M. (2020). NAFLD as a continuum: From obesity to metabolic syndrome and diabetes. Diabetol. Metab. Syndr..

[20] Padda J., Khalid K., Khedr A., Tasnim F., Al-Ewaidat O.A., Cooper A.C., Jean-Charles G. (2021). Non-Alcoholic Fatty Liver Disease and Its Association With Diabetes Mellitus. Cureus.

[21] Singhai A., Yadav V., Joshi R., Malik R.T., Kamle S., Savitha B.T. (2023). Prevalence, Metabolic Profile, and Associated Risk Factors of Non-alcoholic Fatty Liver Disease in an Adult Population of India. Cureus.

[22] Zhou Q., Wang Y., Wang J., Liu Y., Qi D., Yao W., Jiang H., Li T., Huang K., Zhang W. (2021). Prevalence and risk factor analysis for the nonalcoholic fatty liver disease in patients with type 2 diabetes mellitus. Medicine.

[23] Pettinelli P., Fernandez T., Aguirre C., Barrera F., Riquelme A., Fernandez-Verdejo R. (2023). Prevalence of non-alcoholic fatty liver disease and its association with lifestyle habits in adults in Chile: A cross-sectional study from the National Health Survey 2016–2017. Br. J. Nutr..

[24] Walker S.N., Sechrist K.R., Pender N.J. (1987). The Health-Promoting Lifestyle Profile: Development and psychometric characteristics. Nurs. Res..

[25] Walker S.N., Sechrist K.R., Pender N.J. (1995). Health Promotion Model—Instruments to Measure Health Promoting Lifestyle: Health-Promoting Lifestyle Profile [HPLP II] (Adult Version). https://deepblue.lib.umich.edu/handle/2027.2042/85349.

[26] Yang K.C., Liao Y.Y., Tsui P.H., Yeh C.K. (2019). Ultrasound imaging in nonalcoholic liver disease: Current applications and future developments. Quant. Imaging Med. Surg..

[27] Ferraioli G., Soares Monteiro L.B. (2019). Ultrasound-based techniques for the diagnosis of liver steatosis. World J. Gastroenterol..

[28] Saadeh S., Younossi Z.M., Remer E.M., Gramlich T., Ong J.P., Hurley M., Mullen K.D., Cooper J.N., Sheridan M.J. (2002). The utility of radiological imaging in nonalcoholic fatty liver disease. Gastroenterology.

[29] Health Promotion Administration, Ministry of Health and Welfare in Taiwan Metabolic Symdrome. https://www.hpa.gov.tw/pages/list.aspx?nodeid=221.

[30] Pearson T.A., Mensah G.A., Alexander R.W., Anderson J.L., Cannon R.O., Criqui M., Fadl Y.Y., Fortmann S.P., Hong Y., Myers G.L. (2003). Markers of inflammation and cardiovascular disease: Application to clinical and public health practice: A statement for healthcare professionals from the Centers for Disease Control and Prevention and the American Heart Association. Circulation.

[31] Rondina M.T., Weyrich A.S., Zimmerman G.A. (2013). Platelets as cellular effectors of inflammation in vascular diseases. Circ. Res..

[32] Madjid M., Fatemi O. (2013). Components of the complete blood count as risk predictors for coronary heart disease: In-depth review and update. Tex. Heart Inst. J..

[33] Byrne B.M. (2010). Structural Equation Modeling With AMOS: Basic Concepts, Applications, and Programming.

[34] Hair J.F., Black W.C., Babin B.J., Anderson R.E. (2010). Multivariate Data Analysis.

[35] Kline R.B. (2011). Principles and Practice of Structural Equation Modeling.

[36] Le M.H., Yeo Y.H., Li X., Li J., Zou B., Wu Y., Ye Q., Huang D.Q., Zhao C., Zhang J. (2022). 2019 Global NAFLD Prevalence: A Systematic Review and Meta-analysis. Clin. Gastroenterol. Hepatol..

[37] Wang J., Chiu W.H., Chen R.C., Chen F.L., Tung T.H. (2015). The clinical investigation of disparity of nonalcoholic fatty liver disease in a Chinese occupational population in Taipei, Taiwan: Experience at a teaching hospital. Asia Pac. J. Public Health.

[38] Summart U., Thinkhamrop B., Chamadol N., Khuntikeo N., Songthamwat M., Kim C.S. (2017). Gender differences in the prevalence of nonalcoholic fatty liver disease in the Northeast of Thailand: A population-based cross-sectional study. F1000Research.

[39] Salvoza N.C., Claudio P.J., Tiribelli C., Rosso N. (2020). Sex differences in non-alcoholic fatty liver disease: Hints for future management of the disease. Explor. Med..

[40] Pender N.J., Walker S.N., Sechrist K.R., Frank-Stromborg M. (1990). Predicting health-promoting lifestyles in the workplace. Nurs. Res..

[41] Lusk S.L., Kerr M.J., Ronis D.L. (1995). Health-promoting lifestyles of blue-collar, skilled trade, and white-collar workers. Nurs. Res..

[42] Hallsworth K., Adams L.A. (2019). Lifestyle modification in NAFLD/NASH: Facts and figures. JHEP Rep..

[43] Jahromi M.K., Daftari G., Farhadnejad H., Tehrani A.N., Teymoori F., Salehi-Sahlabadi A., Mirmiran P. (2023). The association of healthy lifestyle score and risk of non-alcoholic fatty liver disease. BMC Public Health.

[44] Lu F.B., Hu E.D., Xu L.M., Chen L., Wu J.L., Li H., Chen D.Z., Chen Y.P. (2018). The relationship between obesity and the severity of non-alcoholic fatty liver disease: Systematic review and meta-analysis. Expert Rev. Gastroenterol. Hepatol..

[45] Adams L.A., Anstee Q.M., Tilg H., Targher G. (2017). Non-alcoholic fatty liver disease and its relationship with cardiovascular disease and other extrahepatic diseases. Gut.

[46] Wang C.C., Tseng T.C., Hsieh T.C., Hsu C.S., Wang P.C., Lin H.H., Kao J.H. (2012). Severity of fatty liver on ultrasound correlates with metabolic and cardiovascular risk. Kaohsiung J. Med. Sci..

